# Four-Year Survival Outcomes of Personalized Total Neoadjuvant Therapy Versus Chemotherapy During the ‘Wait Period’ Versus Standard Chemoradiotherapy for Locally Advanced Rectal Cancer

**DOI:** 10.1007/s12029-026-01551-6

**Published:** 2026-07-30

**Authors:** Sergei Bedrikovetski, Zachary Bunjo, Ishraq Murshed, James W. Moore, Tarik Sammour

**Affiliations:** 1 School of Medicine, College of Health, Adelaide University, Adelaide, SA Australia; 2 Colorectal Unit, Department of Surgery, Royal Adelaide Hospital, 5E 334, Port Road, Adelaide, SA 5000 Australia

**Keywords:** Rectal cancer, Total neoadjuvant therapy, Induction chemotherapy, Consolidation chemotherapy, Neoadjuvant chemoradiotherapy, Survival

## Abstract

**Purpose:**

Total Neoadjuvant Therapy (TNT) is increasingly replacing standard chemoradiotherapy (sCRT) for the treatment of locally advanced rectal cancer (LARC). Data on the use of personalized TNT (pTNT) regimens tailored to patient disease characteristics remain scarce. Accordingly, this study aimed to assess long-term outcomes of pTNT in patients with LARC.

**Methods:**

This was a secondary analysis of a multicentre retrospective cohort study. Patients treated with pTNT between 2019 and 2022 were compared to a historical cohort from the WAIT trial (2012–2014), which included extended chemotherapy during the interval period (xCRT) or sCRT followed by adjuvant chemotherapy. The main outcomes were 4-year disease-free survival (DFS) and overall survival (OS).

**Results:**

Forty patients treated with pTNT were matched with 49 patients from the WAIT trial (25 xCRT, 24 sCRT). All WAIT trial patients underwent surgery, whereas 27 (67.5%) pTNT patients underwent surgery and 13 (32.5%) were managed non-operatively. Median follow-up was 48 months. No significant differences were observed in 4-year DFS (pTNT 70% vs. xCRT 68% vs. sCRT 75%, *P* = 0.764) or OS (pTNT 82.5% vs. xCRT 80% vs. sCRT 83.3%, *P* = 0.907). Within the pTNT group, patients with an oCR had significantly higher OS compared to those without oCR (95.2% vs. 68.4%, *P* = 0.021).

**Conclusion:**

Our findings suggest that pTNT achieves comparable survival outcomes to xCRT and sCRT in patients with LARC, despite higher rates of non-operative management. These findings should be interpreted cautiously given the exploratory nature of the analysis and limited sample size. Larger studies with longer follow-up are required to validate these early data.

**Supplementary Information:**

The online version contains supplementary material available at 10.1007/s12029-026-01551-6.

## Introduction

Total neoadjuvant therapy (TNT), incorporating neoadjuvant systemic chemotherapy and chemoradiotherapy before surgical resection or nonoperative management, has emerged as the preferred treatment strategy for locally advanced rectal cancer (LARC) [1]. TNT improves treatment compliance, enhances tumour downstaging, and increases organ preservation rates. However, survival data from TNT trials have shown mixed results, possibly reflecting differences in study design, eligibility criteria, endpoints, and treatment approaches. While an overall survival (OS) benefit was reported in the 7-year results of the PRODIGE-23 trial and the 3-year results of the STELLAR trial, the RAPIDO trial did not show an OS benefit for TNT over standard neoadjuvant chemoradiotherapy (sCRT) [2–4]. 

Although most studies have focused primarily on TNT with either an induction or consolidation chemotherapy approach, recent data support the safe omission of neoadjuvant radiotherapy in selected low-risk tumours, along with tailoring neoadjuvant treatment based on tumour biology (e.g. microsatellite instability-high status) [5, 6]. These developments have contributed to a growing emphasis on personalized treatment strategies for LARC [7, 8]. While selecting induction or consolidation TNT regimens based on disease profile (termed ‘personalized TNT’ or ‘pTNT’) shows promise in improving tumour response and organ preservation in advanced rectal cancer, the survival benefit of a pTNT approach remains unconfirmed [9]. 

We hypothesized that a pTNT approach would increase the proportion of patients achieving an overall complete response (oCR) (defined as clinical complete response [cCR] and/or pathological complete response [pCR]) and ultimately improve disease-free survival (DFS) and OS compared with sCRT alone. Initial results from our multicentre retrospective cohort study demonstrated a significantly higher oCR rate in the pTNT group compared with extended chemotherapy during the ‘wait period’ (xCRT) and sCRT (52.5% versus 24.2% versus 29.2%, *P* = 0.043) [10]. The aim of this secondary analysis was to assess 4-year DFS and OS across the three neoadjuvant treatment groups.

## Methods

### Ethics Approval

The study was performed in line with the principles of the Declaration of Helsinki. Approval was granted by the Central Adelaide Local Health Network (CALHN) (HREC/15/RAH/186) and St. Andrew’s Hospital Human Research Ethics Committee (#117). Given the retrospective nature of this secondary analysis, the requirement for informed consent was waived.

### Study Design

The study design and eligibility criteria have been described in detail elsewhere [10]. In brief, this was a secondary analysis of a multicentre retrospective cohort study comparing oCR in consecutive patients with LARC treated with a pTNT approach over a 3-year period (2019–2022), to a historical cohort of patients treated with xCRT or sCRT as part of the randomized WAIT Trial (2012–2014) [10]. pTNT patients were matched to xCRT and sCRT patients based on the following criteria from the WAIT trial: histologically proven distal rectal carcinoma (defined as being less than 12 cm from the anal verge on rigid sigmoidoscopy), non-metastatic (M0) disease and clinical stage T3/4 or any node-positive disease [11]. 

### Treatment Protocols

Treatment protocols have been described previously [10]. In summary, the pTNT approach consisted of two distinct treatment schemes, in which TNT sequencing was tailored according to patient disease risk based on clinical staging at presentation [9]. Patients with high risk of systemic failure (e.g., extramural vascular invasion [EMVI], mesorectal or lateral pelvic lymph node involvement) received 6–8 cycles of induction chemotherapy before long-course CRT, whereas those at high risk for locoregional failure (e.g., bulky local disease, T4 extension and low tumours) received 6–8 cycles of consolidation chemotherapy after long-course CRT. Chemotherapy regimens consisted of 8 cycles mFOLFOX6 (5-Fluorouracil [5-FU], leucovorin, and oxaliplatin), fortnightly for 16 weeks or 6 cycles of CAPOX (capecitabine and oxaliplatin) for 18 weeks. Long-course CRT involved 50 Gy (Gy) in 25 fractions over 5 weeks with concurrent 5-FU or capecitabine (optional dose escalation to 50.4 Gy in 27 fractions). Response evaluation was assessed within 10 weeks after long-course CRT for induction TNT and within 4 weeks after completion of chemotherapy for consolidation TNT, using computed tomography (CT), flexible sigmoidoscopy, and pelvic magnetic resonance imaging (MRI). Patients with an incomplete clinical response were recommended to undergo surgery, while those who achieved a cCR were offered watch-and-wait (W&W). The W&W surveillance protocol consisted of 3-monthly digital rectal examination, flexible sigmoidoscopy, carcinoembryonic antigen testing and pelvic MRI for the first year, followed by 6-monthly for 5 years. CT scans of the chest, abdomen and pelvis were performed annually for 5 years, and colonoscopy was performed according to Cancer Council Australia guidelines [12]. 

In the WAIT trial, patients on xCRT group received 3 cycles of consolidation chemotherapy following long-course CRT, whereas those on sCRT group only received long-course CRT with no preoperative chemotherapy. Chemotherapy consisted of 3-weekly cycles of bolus 5-FU with leucovorin, administered on each of 3 days per cycle. Long-course CRT involved 45 Gy in 25 fractions over 5 weeks with concurrent 5-FU or capecitabine (optional dose escalation to 50.4 Gy in 28 fractions). Patients were scheduled for surgery 10 weeks after completion of radiotherapy, with adjuvant chemotherapy left to the discretion of the treating team. Patients were not routinely restaged prior to surgery. An examination under anaesthesia was performed prior to surgical resection by a surgeon blinded to treatment allocation, and cCR status was ascertained. At the time of the WAIT trial, no W&W protocol existed for patients who achieved a cCR at participating hospitals.

### Endpoints

The primary endpoint was DFS, defined as the interval from the start of treatment to the first occurrence of local recurrence, distant recurrence, a new colorectal primary cancer or death. Local recurrence was defined as either an unresectable rectal primary tumour following neoadjuvant treatment, an R2 resection for the rectal primary tumour, or recurrence in the primary tumour bed after an R0-R1 resection. Distant recurrence was defined as relapse of the tumour outside the pelvic region. In the pTNT cohort, tumour regrowth in the rectal wall or in regional lymph nodes after cCR and a period of W&W) was not considered local recurrence if it was followed by an R0-R1 resection, as per the OPRA trial [13]. The secondary endpoint was OS, defined as the interval from start of treatment to date of death. Patients without events were censored at the date of last follow-up.

### Statistical Analysis

Statistical analyses were conducted on an intention-to-treat basis. Continuous variable parametricity was tested using the Shapiro-Wilk test, and results were expressed as mean (standard deviation) for parametric data and median (range) for nonparametric data. Continuous variables were compared using one-way ANOVA or the Kruskal-Wallis test depending on the type of distribution. Categorical variables were presented as absolute numbers and percentages. Categorical variables were compared using Pearson’s χ^2^ test or Fisher’s exact test. Kaplan-Meier curves were used to estimate survival, and comparisons between groups were made using the log-rank test. To account for potential immortal time bias introduced by the interval required for treatment completion and cCR assessment, a 6-month landmark analysis was performed for DFS and OS. This analysis included only patients who were alive and disease-free at 6 months from the start of treatment, with survival outcomes calculated from the 6-month landmark time point onward. Sensitivity analyses included a per-protocol approach, and analyses restricted to patients who underwent surgery. Subgroup analyses compared DFS and OS between patients with and without oCR, both within the pTNT group and across groups. Additional subgroup analyses compared DFS and OS between induction and consolidation TNT approaches within the pTNT group. P-values were two-sided and considered significant if < 0.05. Jamovi for Windows version 2.6.44 (The jamovi project, 2025, https://www.jamovi.org) was used for statistical analysis.

## Results

**Fig. 1:**
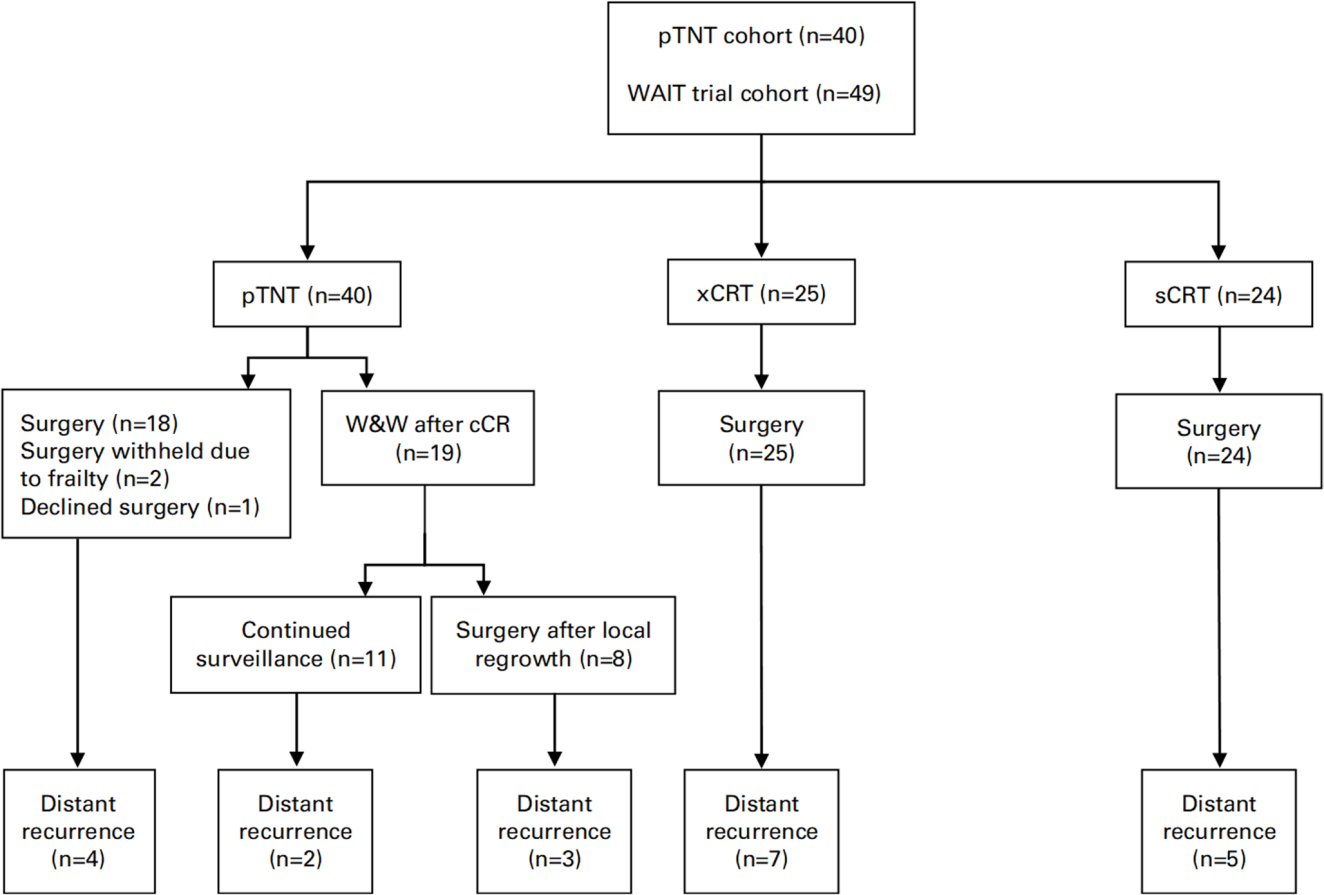
Flowchart illustrating patient allocation, treatment outcomes and follow-up across the three neoadjuvant treatment groups. sCRT, standard neoadjuvant chemoradiotherapy; xCRT, extended chemotherapy during the ‘wait period’; pTNT, personalized total neoadjuvant therapy; W&W, watch-and-wait; cCR, clinical complete response

Forty pTNT patients were matched with 49 WAIT trial patients (25 xCRT, 24 sCRT) per trial eligibility criteria. Baseline patient and tumour characteristics are summarized in Table 1. Patients in the pTNT group had higher rates of cN0 disease (30% vs. 0% vs. 8.3%, *P* < 0.001) and American Society of Anesthesiologists Physical Status 3–4 (69.2% vs. 28% vs. 20.8%, *P* < 0.001) compared with the xCRT and sCRT groups. The median follow-up duration was 48 months, with a mean of 42.72 months (standard deviation 11.52 months). All 49 patients treated with xCRT or sCRT underwent surgery, as mandated by the WAIT trial. Distant recurrence occurred in 5 (20.8%) patients in the sCRT group and 7 (28.0%) patients in the xCRT group (Fig. 1).

**Table 1:** Baseline characteristics

	sCRT (*n* = 24)	xCRT (*n* = 25)	pTNT (*n* = 40)	*P*-value
Age, years (mean ± SD)	60.4 ± 12.5	59.7 ± 10.1	59.2 ± 13.0	0.937
Gender (male: female)	18:6	18:7	26:14	0.970
BMI (kg/m^2^)	26.8 ± 4.3	26.0 ± 3.5	28.8 ± 10.3	0.367
Distance from anal verge (cm)	6.0 ± 2.6	6.6 ± 2.6	5.7 ± 3.0	0.435
Clinical T stage				0.236
T2	1 (4.2)	0 (0.0)	2 (5.0)	
T3	18 (75.0)	24 (96.0)	31 (77.5)	
T4	5 (20.8)	1 (4.0)	7 (17.5)	
Clinical N stage				< 0.001
N0	2 (8.3)	0 (0.0)	12 (30.0)	
N1	7 (29.2)	6 (24.0)	23 (57.5)	
N2	15 (62.5)	19 (76.0)	5 (12.5)	
CRM				0.350
Clear	12 (50.0)	10 (40.0)	15 (37.5)	
Threatened	4 (16.7)	8 (32.0)	6 (15.0)	
Involved	8 (33.3)	7 (28.0)	19 (47.5)	
EMVI				0.676
Positive	8 (33.3)	11 (44.0)	22 (55.0)	
Negative	16 (66.7)	14 (56.0)	18 (45.0)	
ASA Physical Status ^a^				< 0.001
1–2	19 (79.2)	18 (72.0)	8 (30.8)	
3–4	5 (20.8)	7 (28.0)	18 (69.2)	

BMI, body mass index; CRM, circumferential resection margin; EMVI, extramural vascular invasion; ASA, The American Society of Anesthesiologists

^a^ Only includes patients who underwent surgery

In the pTNT group, 19 (47.5%) patients achieved a cCR and entered W&W, 18 (45.0%) underwent surgery, two (5%) were deemed too frail for surgery, and one (2.5%) patient declined surgery and opted for W&W. Among patients who initially achieved a cCR, 8 (42.1%) developed tumour regrowth and underwent salvage surgery, while 11 (57.9%) had a sustained cCR and continued W&W. Distant recurrence occurred in 4 (21.1%) patients undergoing surgery after restaging, 2 patients (18.2%) with sustained cCR, and 3 (37.5%) patients after salvage surgery. No local recurrences were detected in any of the three treatment groups.

**Fig. 2:**
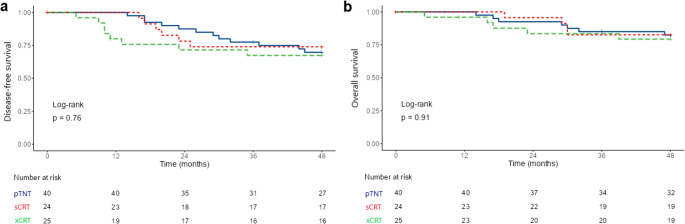
Kaplan-Meier curves showing (**a**) 4-year disease-free survival and (**b**) 4-year overall survival according to neoadjuvant treatment. sCRT, standard neoadjuvant chemoradiotherapy; xCRT, extended chemotherapy during the ‘wait period’; pTNT, personalized total neoadjuvant therapy

**Fig. 3:**
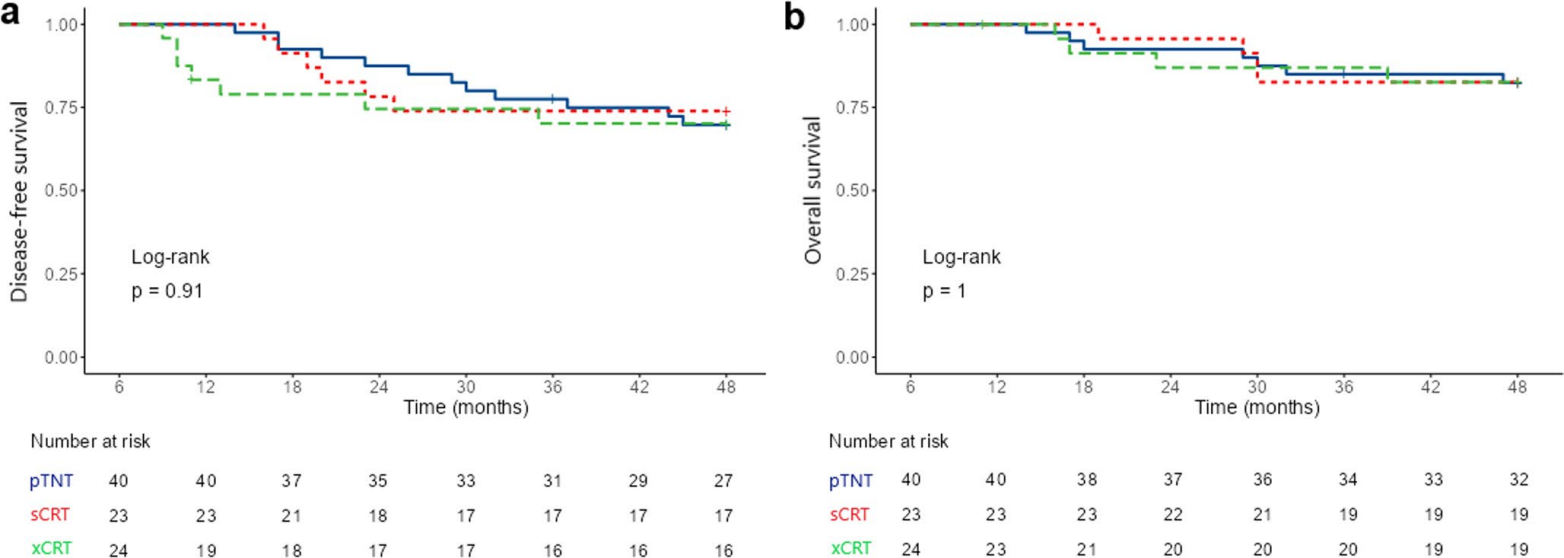
Kaplan-Meier curves showing (**a**) disease-free survival and (**b**) overall survival, landmarked at 6 months from treatment initiation, according to neoadjuvant treatment. sCRT, standard neoadjuvant chemoradiotherapy; xCRT, extended chemotherapy during the ‘wait period’; pTNT, personalized total neoadjuvant therapy

There were no significant differences in 4-year DFS or OS between groups. DFS rates were 69.7% (pTNT), 67.4% (xCRT), and 73.9% (sCRT) (*P* = 0.76; Fig. 2a). OS rates were 82.4%, 79.3%, and 82.6%, respectively (*P* = 0.91; Fig. 2b). DFS events were predominantly due to distant recurrence, accounting for 5 of 6 events in the sCRT group, 7 of 8 in the xCRT group, and 9 of 12 in the pTNT group, with the remaining events due to death from any cause. We implemented a landmark analysis to account for the immortal time bias and included only the patients who lived and were disease-free at least 6 months after the start of treatment in this analysis. Of the 89 patients, two (2.2%) were excluded from the analysis: one patient in the xCRT group died before 6 months and one patient in the sCRT group was lost to follow-up prior to 6 months. DFS rates were 69.7% (pTNT), 70.2% (xCRT) and 73.9% (sCRT) (*P* = 0.91, Fig. 3a). OS rates were 82.4%, 82.6%, 82.6%, respectively (*P* = 1.0, Fig. 3b).

Sensitivity analysis using a per-protocol approach included 37 pTNT, 25 xCRT, and 24 sCRT patients. DFS rates were 70% (pTNT), 67.4% (xCRT), and 73.9% (sCRT) (*P* = 0.76; Supplementary Figure S1a). OS rates were 83.7%, 79.3%, and 82.6%, respectively (*P* = 0.86; Supplementary Figure S1b). Sensitivity analysis restricted to patients who underwent surgery included 26 pTNT patients, 25 xCRT patients and 24 sCRT patients. DFS rates were 65.2% (pTNT), 67.4% (xCRT), and 73.9% (sCRT) (*P* = 0.79; Supplementary Figure S1c). OS rates were 76.7%, 79.3%, and 82.6%, respectively (*P* = 0.88; Supplementary Figure S1d).

**Fig. 4:**
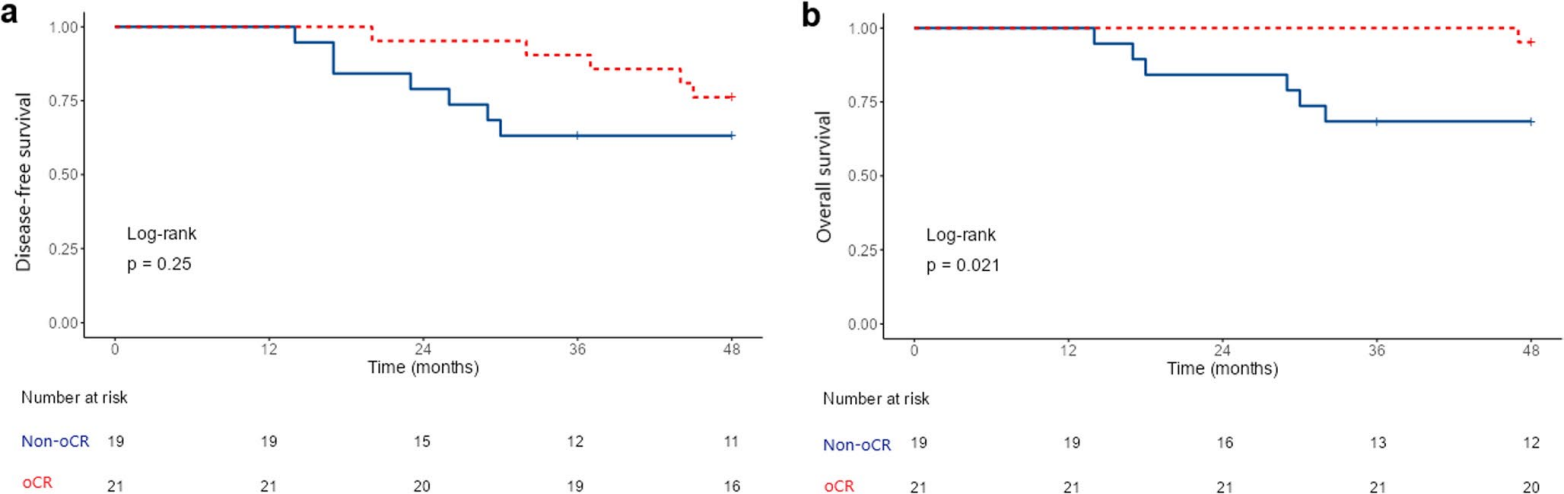
Kaplan-Meier curves showing (**a**) 4-year disease-free survival and (**b**) 4-year overall survival in the pTNT group according to oCR status. sCRT, standard neoadjuvant chemoradiotherapy; xCRT, extended chemotherapy during the ‘wait period’; pTNT, personalized total neoadjuvant therapy; oCR, overall complete response

**Fig. 5:**
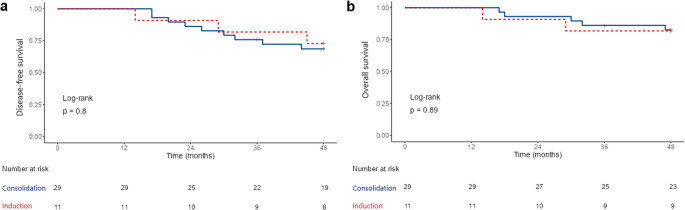
Kaplan-Meier curves showing (**a**) 4-year disease-free survival and (**b**) 4-year overall survival in the pTNT group according to neoadjuvant chemotherapy sequencing

A subgroup analysis comparing DFS and OS between patients with and without oCR is illustrated in Fig. 4 and Supplementary Figure S2. Overall, 21 (52.5%) patients achieved oCR in the pTNT group, compared with 6 (24.2%) in the xCRT group and 7 (29.2%) in the sCRT group. Within the pTNT group, DFS rates were comparable between patients with and without oCR (76.2% vs. 63.2%, *P* = 0.25; Fig. 4a), whereas OS was significantly higher in patients with an oCR compared with those without (95.2% vs. 68.4%, *P* = 0.021; Fig. 4b). Across groups, DFS rates were 76.2% (pTNT), 100% (xCRT), 85.7% (sCRT) for patients with oCR, and 63.2% (pTNT), 57.9% (xCRT), 68.8% (sCRT) for patients without oCR (*P* = 0.29; Supplementary Figure S2a). OS rates were 95.2% (pTNT), 100% (xCRT), 85.7% (sCRT) for patients with oCR and 68.4% (pTNT), 73.7% (xCRT), 81.3% (sCRT) for patients without oCR (*P* = 0.21; Supplementary Figure S2b). A subgroup analysis comparing DFS and OS between induction and consolidation TNT approaches within the pTNT group is shown in Fig. 5. Eleven (27.5%) patients received induction TNT, and 29 (72.5%) received consolidation TNT. DFS rates were 72.7% for induction TNT and 68.6% for consolidation TNT (*P* = 0.80; Fig. 5a), while OS rates were 81.8% and 82.6%, respectively (*P* = 0.89; Fig. 5b).

## Discussion

In this secondary analysis of a multicentre retrospective cohort study, we found comparable 4-year DFS and OS rates across groups, suggesting that pTNT achieves similar long-term oncological outcomes to xCRT and sCRT. This indicates that tailoring TNT sequencing according to individual disease characteristics does not compromise disease control, even when a higher proportion of patients are managed non-operatively. These findings are consistent with previous evidence demonstrating equivalent oncological outcomes between TNT and sCRT [14, 15]. 

Although pTNT nearly doubled the proportion of patients achieving an oCR compared with xCRT and sCRT, this did not translate into improved OS at 4 years. The delivery of 6–8 cycles of neoadjuvant chemotherapy in the pTNT group, compared with three cycles in the xCRT group and none in the sCRT group, reflects a treatment intensification strategy similar to that used in the TIMING trial. In this phase II trial, the addition of consolidation FOLFOX after sCRT significantly increased pCR rates and improved 5-year DFS, but did not confer an OS benefit [16]. The OPRA trial, which examined a planned W&W strategy using TNT, found that higher rates of cCR or near-cCR did not improve DFS compared with historical controls [13]. This may partly reflect differences in post-treatment strategies. Unlike the WAIT and TIMING trials, which mandated surgery following neoadjuvant treatment, both the pTNT cohort and the OPRA trial included patients managed with a W&W strategy after achieving a cCR.

While the PRODIGE 23 trial demonstrated an OS benefit with TNT, this finding has not been consistently reproduced across other randomized studies [17, 18]. The observed benefit may in part reflect differences in outcomes within the control arm, including an unexpectedly early divergence in DFS. Additional factors such as treatment intensification with FOLFIRINOX (5-FU, leucovorin, irinotecan and oxaliplatin) and the high proportion of patients in the TNT arm who received adjuvant chemotherapy (77%) may also have contributed to these results.

In our cohort, 4-year DFS was numerically lower in the pTNT group than in the sCRT group (69.7% vs. 73.9%). As noted by Socha et al., the DFS benefit associated with TNT followed by immediate surgery may be counterbalanced by an increased risk of distant recurrence when a W&W approach is adopted [19]. Consequently, gains in organ preservation may not be fully offset by long-term oncological outcomes. It is also worth noting that, although not statistically significant, the pTNT group included a higher proportion of patients with adverse prognostic features, including EMVI and involved circumferential resection margin (CRM), which may have further influenced DFS outcomes in this cohort.

In a subgroup analysis, we assessed survival outcomes between patients with and without oCR, both within the pTNT group and across neoadjuvant treatment groups. Within the pTNT group, patients with an oCR had a significantly higher 4-year OS compared to those without oCR. This observation is consistent with a secondary analysis of the OPRA trial, which reported improved 3-year OS in patients with cCR compared to those with near-cCR or incomplete response, suggesting that clinical tumour response at restaging has prognostic implications [20]. However, this pattern has not been consistently observed across studies. Dutch and German retrospective multicenter studies have reported no significant differences in 3- and 5-year OS between patients with and without oCR following TNT [21, 22]. As such, this association remains debated and requires substantiation through prospective trials.

Our subgroup analysis comparing induction and consolidation TNT approaches showed similar survival outcomes, consistent with a pooled analysis of the CAO/ARO/AIO-12 and OPRA trials demonstrating equivalent DFS and OS between the two TNT sequences [23]. Another pooled analysis of these trials did not identify any patient subgroups that benefited significantly from one TNT sequence over the other [24]. However, this analysis included a limited range of baseline clinical and tumour characteristics, omitting key prognostic factors such as EMVI, CRM involvement, tumour diameter and sarcopenia status [25–27]. Therefore, we propose that the choice of TNT sequence should be guided by tumour characteristics and the overarching goal of optimizing both survival outcomes and quality of life. In line with our pTNT protocol, emerging consensus suggests that consolidation TNT may be preferred for patients prioritizing locoregional control and potentially higher oCR rates, whereas induction TNT remains a valuable strategy for addressing the risk of distant failure [28]. 

The principal limitation of this study is the small sample size, which may have limited statistical power to detect significant survival differences and precluded the use of robust propensity score-based methods for confounding adjustment. A formal a priori power calculation was not performed, as this was a retrospective study with a fixed sample size determined by available patients. Consequently, between-group differences should be interpreted with caution and non-significant findings should not be interpreted as evidence of equivalence. The 8-year gap between cohorts is another limitation, as the non-contemporaneous cohorts (xCRT and sCRT) may introduce period effects related to advances in surgery, radiological imaging, chemotherapy, radiotherapy and supportive care. Furthermore, the historical WAIT trial cohort underwent mandatory surgery and therefore had no opportunity for organ preservation irrespective of treatment response, limiting comparability with the contemporary pTNT cohort despite a sensitivity analysis restricted to surgically managed patients. Lastly, patients in the W&W pathway must survive long enough to achieve a cCR, potentially introducing immortal time bias that may favour the pTNT group in survival comparisons. This was addressed by performing a 6-month landmark analysis, which mitigates this bias by excluding early events and aligning risk sets across groups. Nevertheless, this is the first study to compare survival outcomes of a pTNT strategy with xCRT and sCRT within the context of a mature W&W protocol. Continued follow-up to 7 years may provide additional insight into long-term outcomes.

## Conclusion

Our findings suggest that pTNT achieves survival outcomes comparable to those of xCRT and sCRT in patients with LARC. Although oCR was associated with improved OS within the pTNT cohort, this finding should be interpreted cautiously given the exploratory nature of the analysis and limited sample size. Despite higher oCR rates with pTNT, this short-term surrogate endpoint did not translate into improved long-term oncological outcomes, underscoring the limitations of cCR and pCR as predictors of survival in rectal cancer.

## Supplementary Information

Below is the link to the electronic supplementary material.

Supplementary Material 1 (PDF 164 KB)

## Data Availability

The data analysed during the current study are not publicly available due to patient data protection regulations and institutional policies but are available from the corresponding author on reasonable request.

## Abbreviations

TNT: Total Neoadjuvant Therapy
pTNT: personalized Total Neoadjuvant Therapy
sCRT: standard Chemoradiotherapy
xCRT: extended chemotherapy during the interval period
LARC: Locally Advanced Rectal Cancer
OS: Overall survival
DFS: Disease-free Survival
oCR: overall Complete Response
cCR: clinical Complete Response
pCR: pathological Complete Response
CALHN: Central Adelaide Local Health Network
EMVI: Extramural Vascular Invasion
Gy: Gray
5-FU: 5-Fluorouracil
mFOLFOX6: modified 5-Fluorouracil Leucovorin Oxaliplatin
CAPOX: Capecitabine Oxaliplatin
FOLFIRINOX: 5-Fluorouracil Leucovorin Irinotecan Oxaliplatin
CT: Computed Tomography
MRI: Magnetic Resonance Imaging
W&W: Watch-and-Wait
