# Supplementary material for: Four-Year Survival Outcomes of Personalized Total Neoadjuvant Therapy Versus Chemotherapy During the ‘Wait Period’ Versus Standard Chemoradiotherapy for Locally Advanced Rectal Cancer
Source: J Gastrointest Cancer. 2026 Jul 30;57(1):171. doi: 10.1007/s12029-026-01551-6 (PMC13424804; doi:10.1007/s12029-026-01551-6)
Supplement: Supplementary file 1 — Supplementary Material 1 (PDF 164 KB) [file 12029_2026_1551_MOESM1_ESM.pdf]

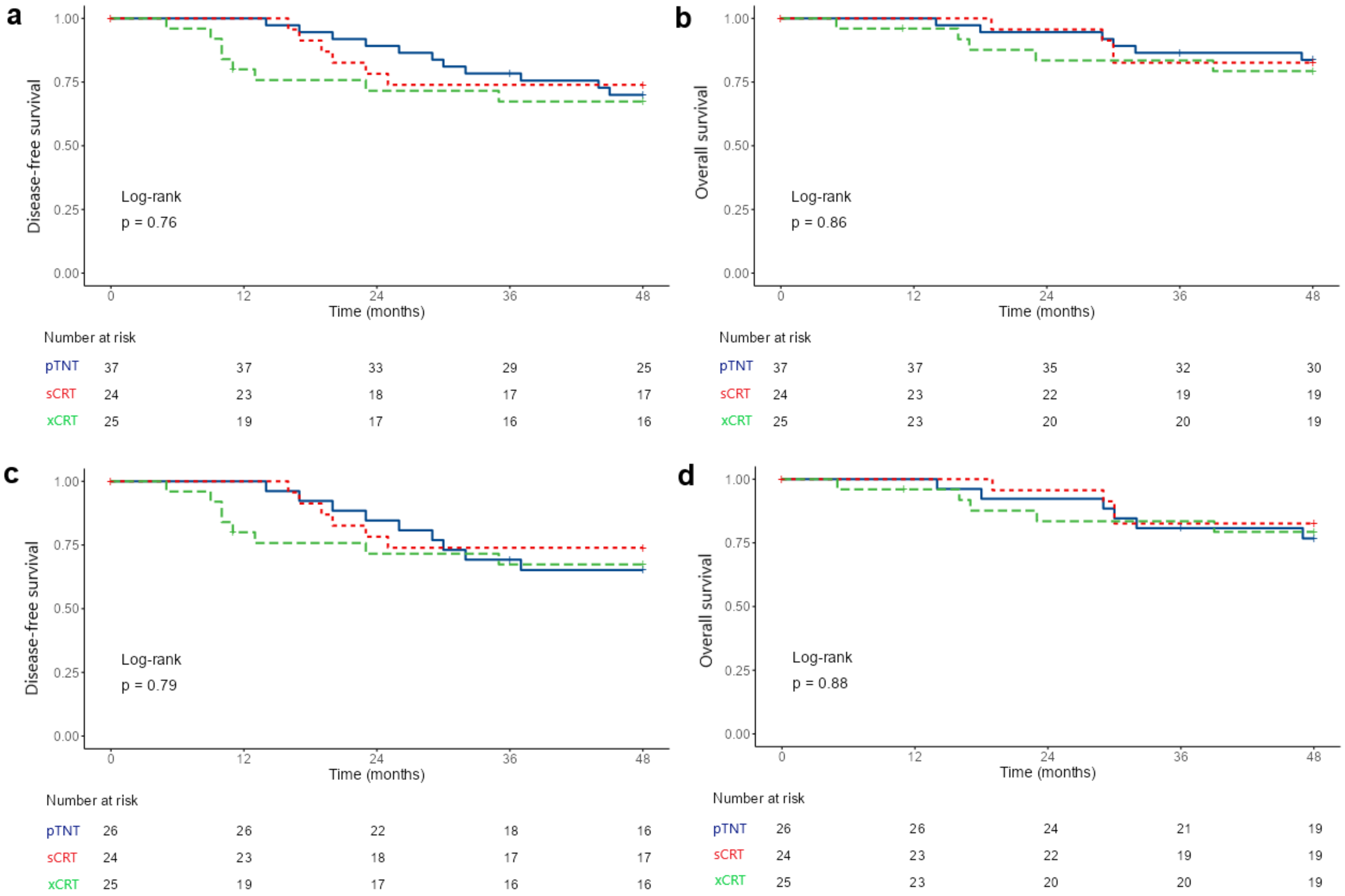

Supplementary Figure S1. Kaplan-Meier curves showing (a) 4-year disease-free survival and (b) 4-year overall survival in the per-protocol population according to neoadjuvant treatment. Kaplan-Meier curves showing (c) 4-year disease-free survival and (d) 4-year overall survival for patients who underwent surgery. sCRT, standard neoadjuvant chemoradiotherapy; xCRT, extended chemotherapy during the ‘wait period’; pTNT, personalized total neoadjuvant therapy.

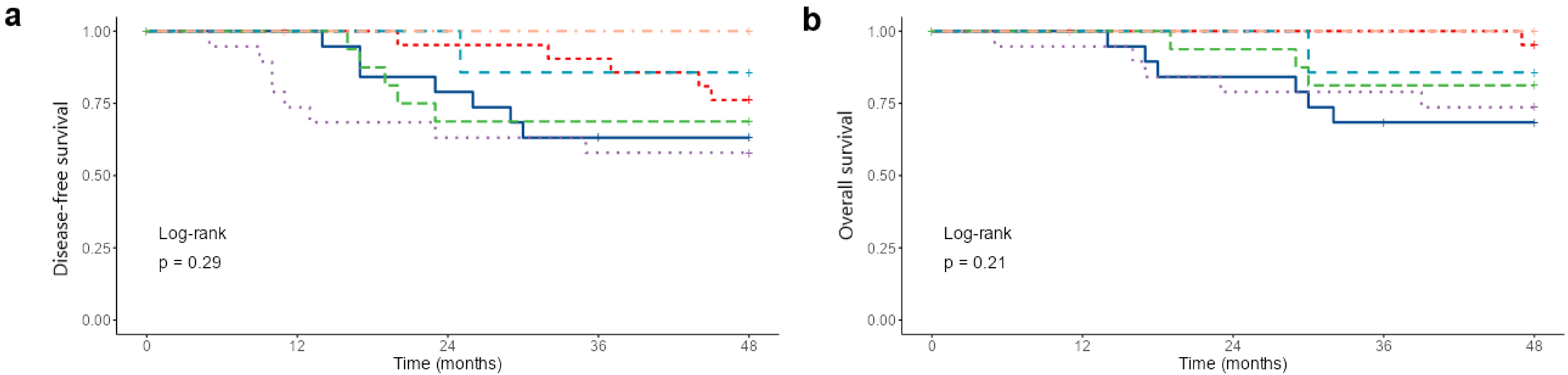

Number at risk (a)

	0	12	24	36	48
pTNT_Non-oCR	19	19	15	12	11
pTNT_oCR	21	21	20	19	16
sCRT_Non-oCR	17	16	11	11	11
sCRT_oCR	7	7	7	6	6
xCRT_Non-oCR	19	14	12	11	11
xCRT_oCR	6	5	5	5	5

Number at risk (b)

	0	12	24	36	48
pTNT_Non-oCR	19	19	16	13	12
pTNT_oCR	21	21	21	21	20
sCRT_Non-oCR	17	16	15	13	13
sCRT_oCR	7	7	7	6	6
xCRT_Non-oCR	19	18	15	15	14
xCRT_oCR	6	5	5	5	5

Supplementary Figure S2. Kaplan-Meier curves showing (a) 4-year disease-free survival and (b) 4-year overall survival according to neoadjuvant treatment and oCR status.
